# Repeatability of individual migration routes, wintering sites, and timing in a long‐distance migrant bird

**DOI:** 10.1002/ece3.2578

**Published:** 2016-11-11

**Authors:** Rien E. van Wijk, Silke Bauer, Michael Schaub

**Affiliations:** ^1^ Swiss Ornithological InstituteSempachSwitzerland; ^2^ Department of Evolutionary Biology and Environmental StudiesUniversity of ZürichZürichSwitzerland; ^3^ Institute of Ecology and EvolutionDivision of Conservation BiologyUniversity of BernBernSwitzerland; ^4^ Present address: Rien E. van Wijk Calle Zafra 12 2‐418010GranadaSpain

**Keywords:** autumn migration, first‐time migrants, geolocators, Hoopoes, non‐breeding, Sahel, spring migration

## Abstract

Migratory birds are often faithful to wintering (nonbreeding) sites, and also migration timing is usually remarkably consistent, that is, highly repeatable. Spatiotemporal repeatability can be of advantage for multiple reasons, including familiarity with local resources and predators as well as avoiding the costs of finding a new place, for example, nesting grounds. However, when the environment is variable in space and time, variable site selection and timing might be more rewarding. To date, studies on spatial and temporal repeatability in short‐lived long‐distance migrants are scarce, most notably of first‐time and subsequent migrations. Here, we investigated repeatability in autumn migration directions, wintering sites, and annual migration timing in Hoopoes (*Upupa epops*), a long‐distance migrant, using repeated tracks of adult and first‐time migrants. Even though autumn migration directions were mostly the same, individual wintering sites often changed from year to year with distances between wintering sites exceeding 1,000 km. The timing of migration was repeatable within an individual during autumn, but not during spring migration. We suggest that Hoopoes respond to variable environmental conditions such as north–south shifts in rainfall during winter and differing onset of the food availability during spring migration.

## 1 Introduction

Many bird species are faithful to their breeding grounds (Greenwood, 1980). Site fidelity can be advantageous because of familiarity with local resources and predators (Greenwood, 1980; Part, 1991; Piper, 2011) while searching for a new site may cost energy and increase predation risk (Yoder, Marschall, & Swanson, 2004). In migratory birds, site fidelity may not be restricted to the breeding grounds, but can also apply to wintering (nonbreeding) and stopover sites (López‐López, García‐Ripollés, & Urios, 2014; Senner, Hochachka, Fox, & Afanasyev, 2014; Vardanis, Klaassen, Strandberg, & Alerstam, 2011; Yamamoto et al., 2014). Migrants can also show consistent annual timing, for example, a high repeatability of arrival time in the wintering grounds, especially when site fidelity is high (Conklin, Battley, & Potter, 2013; Vardanis et al., 2011). Birds that adopt such spatially and temporally repeatable migrations often depend on particular resources that are exclusively available at specific places and times. For example, shorebirds depend on food‐rich mudflats, seabirds follow sea currents, and raptors need specific wind and thermal conditions for migratory progression (Berthold, 2001; Newton, 2008).

Contrastingly, other migrating species rely on resources whose availability varies over time and space such as insects (Andersson, 1980; Schlossberg, 2009). Their best strategy would be to adopt a temporally and spatially flexible migration strategy responding to environmental conditions during migration and in the choice of stationary sites. Many (near) passerines are suspected to use such a flexible strategy as suitable stopover and wintering sites seem to be numerous, yet empirical evidence is scarce. Catry et al. (2004) showed for long‐distance migrant passerines stopping over in spring that site fidelity was low, probably caused by flexible strategies to respond to wind drift during migration, thus making birds end up on different stopover sites each year. Similarly, Stanley, MacPherson, Fraser, McKinnon, and Stutchbury (2012) showed for Wood thrushes (*Hylocichla mustelina*) that both autumn and spring migration routes differed within individuals from year to year, likely due to changes in weather. However, none of these studies have investigated both timing and migration routes over the course of an annual cycle.

In addition to differences in migration routes and timing in response to environmental variability, experience can also alter migratory behavior (Rotics et al., 2016), but to date, differences between first‐time migrants and adults have not been investigated in short‐lived long‐distance migrants.

In this study, we investigated the repeatability of autumn migration directions, migration timing in autumn and spring, and the location of wintering sites in subsequent migrations in a near‐passerine: the Hoopoe (*Upupa epops*) using geolocator data of both first‐time and adult migrants.

## 2 Materials and Methods

### 2.1 Study site and data collection

Our study was conducted in a population of Hoopoes in southern Switzerland (46°14′N 7°22′E) that breed from roughly May until July with up to three broods (Hoffmann, Postma, & Schaub, 2015). Prior to our study, ~700 nestboxes were installed in the roughly 62 km^2^ study area (Arlettaz, Schaub, Fournier, & Reichlin, 2010). To investigate autumn migration directions, migration timing, and wintering sites, birds were equipped with geolocators of type SOI‐GDL1 (Bächler et al., 2010). These geolocators weighed <2% of the body weight of a Hoopoe and were shown not to affect body condition, physiological state, breeding success, or annual apparent survival (van Wijk, Souchay, Jenni‐Eiermann, Bauer, & Schaub, 2016). Between 2009 and 2014, 328 breeding birds and 459 nestlings were equipped with such geolocators. We retrieved geolocators in the breeding grounds and obtained migration data from 10 adults over 2 years, two adults over 3 years, and six‐first‐time migrants (“juveniles”) during their first and subsequent migration.

### 2.2 Migration data

Geolocator data were analyzed as described in detail in van Wijk et al. (2016). This procedure used the *TrendLight* function in R (Schmaljohann et al., 2015) to define stationary periods, which were required to describe the (1) departure date from the breeding grounds; (2) arrival date in the wintering sites; (3) departure date from the wintering sites; (4) arrival date in the breeding grounds; and (5) the duration of autumn and spring migration. The *TrendLight* function uses both *GeoLight* (Lisovski & Hahn, 2012) and a derivative of *mergesites* (following Liechti et al., 2015) that successively merges periods when the modus of the next site location does not differ by more than 200 km and both sites were stationary. The wintering site was then defined as the first site where birds stayed for at least 6 weeks after leaving the breeding grounds, and their positions were calculated for the period November until January (middle of the wintering period; “midwinter”) to avoid influence of the equinox and to keep the method comparable between birds. On the few occasions that birds used two wintering sites, the position of the first site was calculated from the 1st of November until departure and the position of the second site from arrival until 31st of January. We calculated positions using site‐specific sun elevation angles for each wintering site (varying between −3 and −7) using the HillEkstrom method within the R package *GeoLight* (Ekstrom, 2004; Hill, 1994; Lisovski & Hahn, 2012). From the combined final positions derived from the geolocator data midwinter for each wintering site, kernel densities were calculated using ArcGIS (Silverman, 1986) and the 25% kernel used for graphical presentations. For one adult, we lacked sufficient positions in winter, likely because of temporary battery failure that resulted in data gaps and unnatural sun events.

Autumn migration directions were determined using longitudinal data after departure from the breeding grounds, and we assessed whether birds used a route via (1) the Iberian Peninsula; (2) mainland Italy; or (3) islands in the western Mediterranean Sea. We used this coarse classification of autumn migration directions to overcome problems with positioning during equinox. Spring migration routes were not investigated. There were insufficient data to retrace the birds’ migratory routes in spring, because some geolocators had ceased to record prior to spring.

### 2.3 Data analysis

To assess the spatial scale at which individuals had used the same (or different) wintering sites, we calculated the great circle distance between the modi of all wintering positions. Because of the uncertainty of geolocator position estimates (Lisovski et al., 2012), we assumed that a bird had changed wintering location when the distance between wintering sites was larger than 150 or, more conservatively, 250 km. Directions of site shifts were visualized using Oriana 4.

To test the repeatability of migration timing, we used a linear mixed‐effects model implemented in the R package *rtpR* (Nakagawa & Schielzeth, 2010). We used the restricted maximum‐likelihood (REML) estimation of repeatability for largely unbiased estimates of variance. Repeatability is a product of the variance between and within individuals (Bell, Hankison, & Laskowski, 2009; Conklin et al., 2013). To highlight the within‐individual variability, we used the median absolute difference in timing between years: lower values indicate lower within‐individual variability. As the timing of adult and first‐time migrants and the timing between sexes are very similar in Hoopoes (R. E. van Wijk, unpublished data), we combined data of both sex and age classes to increase sample size.

## 3 Results

**Figure 1:**
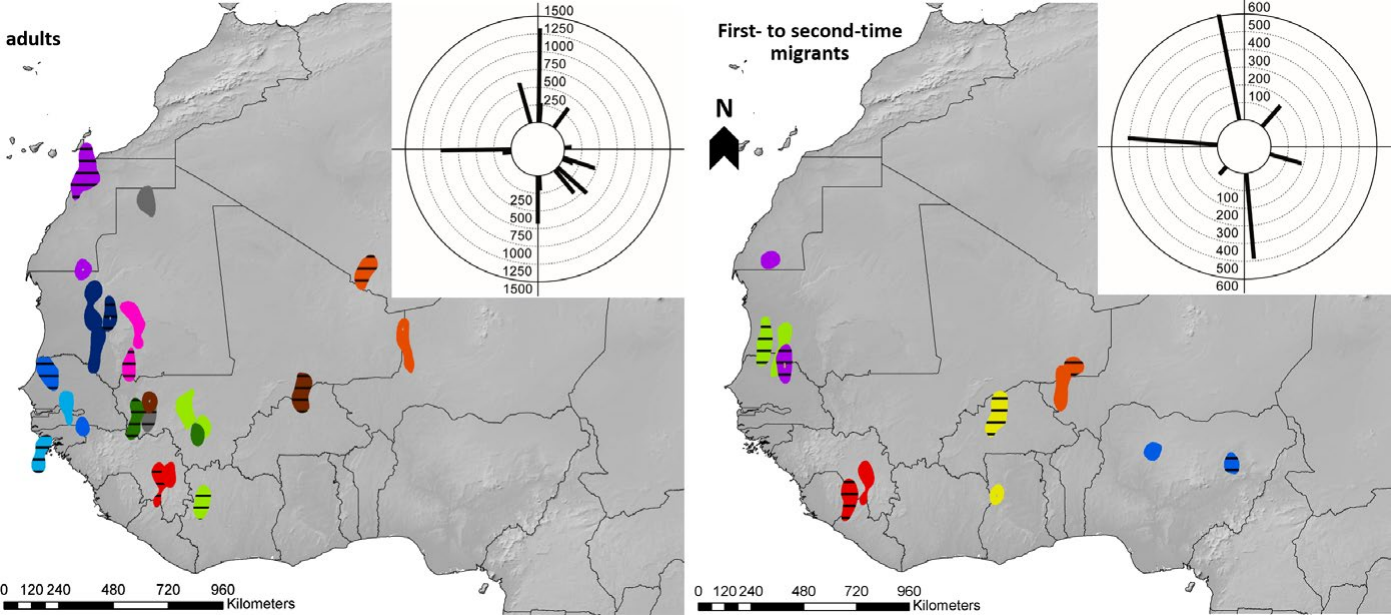
Wintering sites of adult and first‐time migrants as represented by 25% kernels based on position data for the months November until January. In cases of multiple wintering sites, only the first is shown. Each color patch represents the wintering site of one individual (see Table 1) with barred patterns referring to the wintering sites in the first migration recorded. Insets show the directions combined with relative distance (in kilometers) of wintering site shifts between years

Both adult and first‐time migrants spread over a vast area throughout western Africa (~2,000 km west–east corresponding to an area of 1.7 million km^2^ (adults) and 2.1 million km^2^ (first‐time migrants), Figure 1), and the majority of birds used the same autumn migration direction each year. In adults, roughly 69% followed the same direction (*N* = 12) and 75% of first‐time migrants repeated their direction as adult migrants (*N* = 4, Table 1). Individuals that changed autumn migration directions (*N* = 5) usually switched to a faster route via islands in the western Mediterranean Sea (80% of cases; Table 1, cf. van Wijk et al., 2016).

**Table 1:** Overview of individuals in the analysis on spatial repeatability of migration indicating which autumn migration directions were used in the first (y1) and subsequent years (y2, y3) of tracking; the distances between wintering sites (rounded to the nearest 25 km); the number of wintering sites in a season; and which color corresponds to each individual in Figure 1

Age	Sex	Year	Ring	Autumn migration directions			Site distances (km)		No. of wintering sites			Color Figure 1
				y1	y2	y3	1–2	2–3	y1	y2	y3	
Adults	M	2009	H107445	Iberia	Iberia	Iberia	575	125	1	1	1	Light green
	F	2009	H107459	Islands	Iberia	Islands	100	175	1	1	1	Dark blue
	M	2009	H107582	Iberia	Iberia		450		2	2		Dark green
	M	2010	H110718	Iberia	Iberia		250		1	1		Pink
	F	2010	H110911	Iberia	Iberia		1,300		1	1		Gray
	F	2011	H111115	Iberia	Iberia		400		1	1		Blue
	F	2011	H111176	Iberia	Islands		325		1	1		Light blue
	M	2011	H115004	Iberia	–		650		1	1		Purple
	M	2011	H77452	Islands	Islands		975		1	1		Brown
	F	2012	H117910	Iberia	Islands		525		1	2		Orange
	F	2012	H117873	Islands	Islands		1,125		–	–		–
	M	2013	H44866	Iberia	Iberia		75		1	1		Red
Juveniles	F	2012	H102456	Islands	Islands		175		1	1		Green
	M	2012	H117732	–	Islands		500		1	1		Blue
	M	2012	H44866	–	Iberia		150		1	1		Red
	F	2013	H117935	Islands	Islands		475		1	1		Yellow
	F	2013	H121128	Iberia	Iberia		600		1	1		Purple
	F	2013	H121752	Italy	Islands		50		1	1		Orange

Autumn migration directions were either via the Iberian Peninsula (Iberia), islands between the Iberian Peninsula and mainland Italy (Islands) or mainland Italy (Italy). – indicates that no data were available. Age refers to the age at the first autumn migration.

Using a threshold of 150 km, 21% of the adults and 33% of the first‐time migrants visited the same wintering sites in successive years, whereas for the more conservative 250 km’ threshold, this was 36% vs. 50% (Table 1).

The distances between wintering sites did not differ between adults and first‐time migrants (Kruskal–Wallis rank‐sum test *p* > .05; median of 319 ± interquartile range of 329 km in first‐time migrants, *N* = 6 vs. 411 ± 404 km, *N* = 13 in adults, see also Figure 1), and also the directions of shifts in winter sites did not follow a prevalent direction for both adults and first‐time migrants (Figure 1, Moore's modified Rayleigh test *p* > .05).

**Figure 2:**
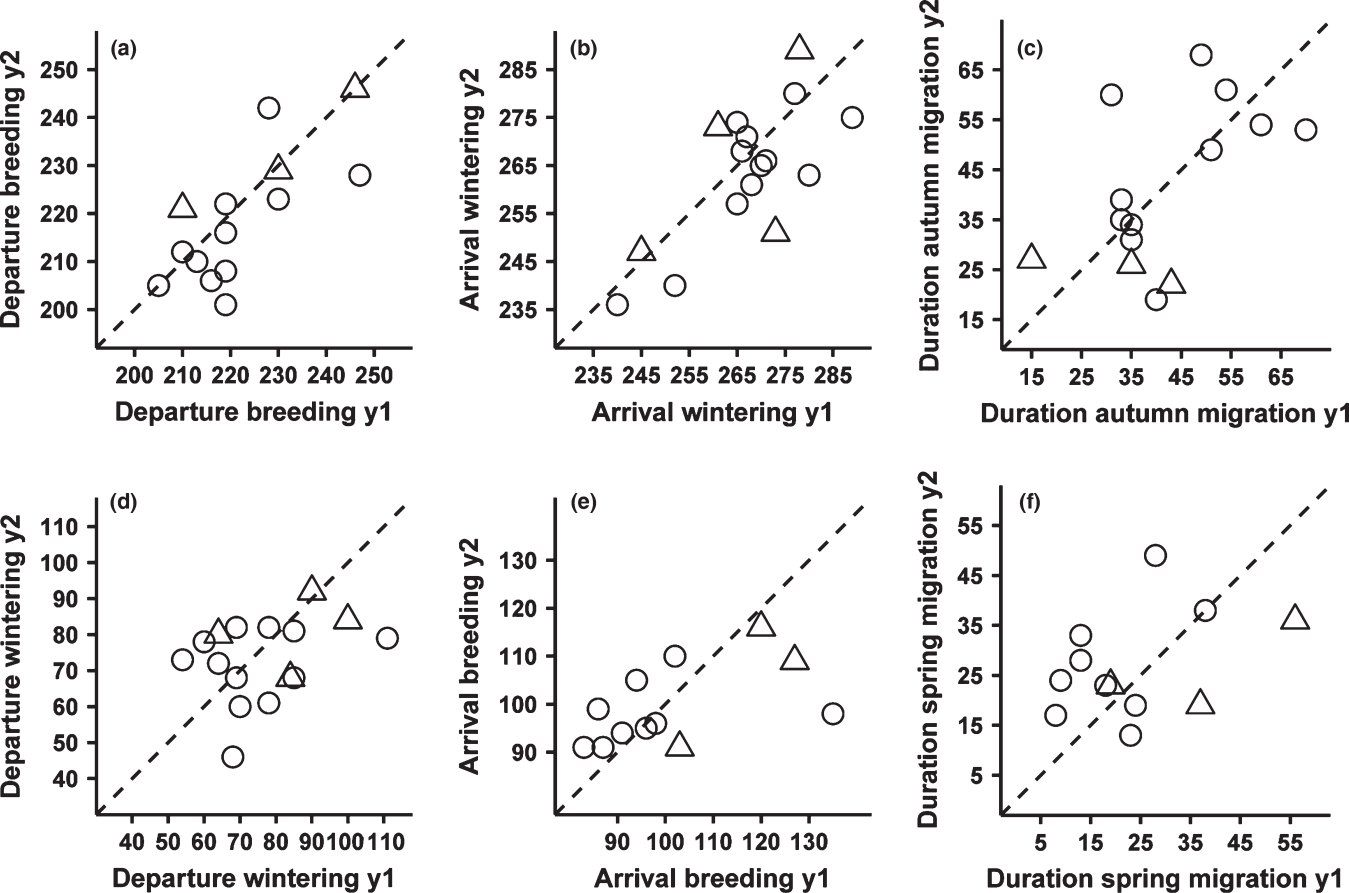
Repeatability of timing between successive individual migration bouts for adult (circles) and first‐time migrants (triangles). The dashed line represents the highest achievable repeatability score of 1, that is, when individuals would have used the same timing in successive migrations. Dates are given as Julian days with the 1st of January being day 1, durations in number of days

Timing of migration was highly repeatable and significantly different from zero for departure from the breeding grounds and arrival in the wintering sites, and moderately repeatable for the duration of autumn migration (Figure 2a–c, Table 2). In contrast, repeatability was low and not significantly different from zero for departure from the wintering sites and arrival in the breeding grounds (Figure 2d,e, Table 2), and consequently also for the duration of spring migration (Figure 2f, Table 2).

**Table 2:** Repeatability (*R*) with 95% confidence interval (CI) for each timing event (calculated from the absolute difference in timing between years in median number of days). Given is also sample size (*N*, number of individuals) and a significance test whether *R* is larger than zero (*p*)

Migration event	*N*	Repeatability			Within individuals variability
		*R*	CI	*p*	
Departure breeding	14	.75	0.42–0.91	<.01	5.0 days
Arrival wintering	16	.73	0.41–0.89	<.01	7.5 days
Duration autumn	14	.51	<0.001–0.80	.03	8 days
Departure wintering	16	.24	0–0.64	.17	16 days
Arrival breeding	12	.43	0–0.83	.07	8 days
Duration spring	12	.21	0–0.66	.21	12.5 days

## 4 Discussion

We found that the majority of both adult and first‐time migrants changed wintering sites from year to year despite following consistent autumn migration directions. Repeatability of timing was high for autumn, but not for spring migration.

Autumn migration directions were mostly the same between years in both first‐time and adult migrants, suggesting that after their first migration, individuals repeat the general route that had been proven successful in the previous year. Changes of direction might relate to increasing experience (Rotics et al., 2016) or might have been caused by different environmental circumstances, for example, wind conditions (Liechti, 2006). As directions were similar between years for both first‐time migrants and adults, it seems likely that differing environmental conditions played a role. We deliberately chose the term “directions”, because we did not investigate finer‐scale movements, for example, specific stopover site use. The exact location of stopovers may have changed between years in which case consistency would have been lower (Catry et al., 2004).

Even though most birds appear to have changed wintering sites between years, this highly depended on the spatial scale, that is, the threshold that defined a site change. In general, Hoopoes showed fidelity to a general region, but apparently not to specific sites. This is in contrast to species that are known to be territorial during winter and which return to almost the same spot each year (Blackburn & Cresswell, 2015; Salewski, Bairlein, & Leisler, 2002). The choice of exact wintering sites can likely be explained by a combination of environmental factors such as food availability and conspecific density. For instance, environmental conditions in West Africa vary greatly from year to year, predominantly under the influence of the Intertropical Convergence Zone (ITCZ or ITF, Intertropical Front; Lélé & Lamb, 2010). Depending on the northward spread of the ITCZ, precipitation varies similarly in a north–southerly direction up to several hundreds of kilometers between years (Lélé & Lamb, 2010). Therefore, birds may be able to profit from occasionally available habitats, such as wadis (Giradoux, Degauquier, Jones, Weigel, & Isenmann, 1988; Hall, 1976), whereas in other years, they have to migrate further south to find suitable habitat. A preliminary exploration of our data with environmental data suggests this might apply to Hoopoes (Table S1); however, we need a larger sample size and/or more fine‐scale data (a higher spatial resolution) for an in‐depth test of the role of environmental variables in the choice of wintering sites.

Additionally, in some years, outbreaks of desert locusts (*Schistocerca gregaria*) may occur, especially in the northern region of the Sahel zone in West Africa (Lecoq, 1978; Tratalos, Cheke, Healey, & Stenseth, 2010). Locusts are known to be an important food source for wintering migrants (Stoate, 1995; Trierweiler et al., 2013), yet their outbreaks are hard to predict (Tratalos & Cheke, 2006). Although the exact diet of Hoopoes in winter is unknown, their typical diet includes large soil invertebrates (e.g., mole crickets on their breeding grounds; Fournier and Arlettaz 2001), suggesting that they could include locusts in their diet if these were available.

Compared to adults, first‐time migrants seem to have spread out over a larger wintering area. First‐time migrants might have responded differently to environmental conditions upon approaching their wintering site. For instance, wind drift might have caused them to spread over larger areas (Thorup, Alerstam, Hake, & Kjellén, 2003) and they might still be inexperienced as to habitat choice (Battley, 2011). As a consequence, they might have ended up in other, suboptimal habitats compared to adult migrants. A larger spread of wintering sites may also result from an active exploration behavior whereby first‐time migrants end up in different sites, further apart, compared to adult migrants (Battley, 2011). In the long‐term, this might be advantageous because first‐time migrants can identify new suitable sites (and thereby, e.g., increase carrying capacity of the population). This hypothesis has been termed the serial residency hypothesis (Cresswell, 2014). However, it assumes high fidelity to wintering sites, which was relatively low in Hoopoes. The hypothesis could be valid in the sense that Hoopoes are faithful to certain regions or habitats, yet whether it fully applies to Hoopoes would also need more detailed data on individual wintering location, habitat use, and foraging behavior.

The few existing studies on the repeatability of migration timing have mostly found that the timing of spring migration was more repeatable than the timing of autumn migration (Stanley et al., 2012; López‐López et al., 2014; but see Alerstam, Hake, & Kjellén, 2006). In contrast, we found the timing of autumn migration to be highly repeatable, but spring migration timing was not. As (selective) pressure is probably less strong for arrival in the wintering grounds than for arrival in the breeding grounds, birds migrate slower in autumn, spending more time on stopovers (Alerstam et al., 2006; Nilsson, Klaassen, & Alerstam, 2013). Consequently, environmental conditions hardly affect the duration of autumn migration (Jenni & Kéry, 2003; Pulido & Widmer, 2005; Schaub & Jenni, 2001), which rather seems to be under endogenous control. In contrast, spring migration is much more influenced by the environment (Balbontín et al., 2009; Both, Bijlsma, & Visser, 2005; Hüppop & Hüppop, 2003; Sokolov & Kosarev, 2003). The exact departure date from the wintering sites thus likely varies from year to year depending on the local conditions, after which birds try to migrate as quickly as possible back to the breeding grounds to increase reproductive success, as seems to be the case in Hoopoes as well (van Wijk, Schaub, & Bauer, in press). Meanwhile, individuals may have to adapt their timing between years to cope with environmental variability *en route* (Balbontín et al., 2009; Bauer, Gienapp, & Madsen, 2008). The differences in timing in response to yearly differences in environmental conditions will cause apparent low repeatability of timing within an individual during spring. The differences in selective pressures on timing and corresponding responses to environmental changes between autumn and spring likely explain the difference in repeatability of timing we found.

Although our sample size was relatively low, we believe that our results provide a good first glimpse on repeatability of migration over the annual cycle in short‐lived, long‐distance migrants. However, the small sample size prevented more thorough assessments of possible ecological reasons for the patterns that we describe, something that deserves future study with more (accurate) data. From our data, we conclude that Hoopoes overall seem very flexible in spring migration timing and the choice of wintering sites and propose that they could be classified as opportunistic migrants.

## Conflict of Interest

None declared.
