# Supplementary material for: Repeatability of individual migration routes, wintering sites, and timing in a long‐distance migrant bird
Source: Ecol Evol. 2016 Nov 11;6(24):8679–85. doi: 10.1002/ece3.2578 (PMC5192954; doi:10.1002/ece3.2578)
Supplement: Supplementary file 1 [file ECE3-6-8679-s001.docx]

**Appendix 1: wintering sites and their environmental characteristics as depicted by NDVI**

Table S1. Expansion of table 1 from the main text including the median NDVI values of the wintering location over the period October until February and the range in NDVI values of the same site for the same period (maximum NDVI values minus the minimum NDVI value). The median NDVI is a proxy for the general greenness of the area, whereas the range indicates how variable the habitat was: the more negative the value, the bigger the difference between the beginning and the end of the wintering period.

| age | sex | year | ring | NDVI median | | | ΔNDVI | | | colour Fig. 1 |
| --- | --- | --- | --- | --- | --- | --- | --- | --- | --- | --- |
|  |  |  |  | *y1* | *y2* | *y3* | *y1* | *y2* | *y3* |  |
| adults | M | 2009 | H107445 | - | 0.22 | 0.25 | - | -0.032 | -0.021 | Light green |
|  | F | 2009 | H107459 | 0.31 | 0.22 |  | -0.028 | -0.023 |  | Dark blue |
|  | M | 2009 | H107582 | 0.34 | 0.19 |  | -0.032 | -0.013 |  | Dark green |
|  | M | 2010 | H110718 | 0.19 | 0.13 |  | -0.013 | -0.002 |  | Pink |
|  | F | 2010 | H110911 | 0.50 | - |  | -0.031 | - |  | Grey |
|  | F | 2011 | H111115 | 0.16 | 0.21 |  | -0.018 | -0.012 |  | Blue |
|  | F | 2011 | H111176 | 0.15 | - |  | -0.001 | - |  | Light blue |
|  | M | 2011 | H115004 | 0.17 | - |  | -0.012 | - |  | Purple |
|  | M | 2011 | H77452 | 0.15 | 0.20 |  | -0.026 | -0.015 |  | Brown |
|  | F | 2012 | H117910 | 0.13 | - |  | -0.001 | - |  | Orange |
|  | F | 2012 | H117873 |  |  |  |  |  |  | - |
|  | M | 2013 | H44866 | 0.53 | 0.53 |  | -0.031 | -0.028 |  | Red |
| juveniles | F | 2012 | H102456 | 0.15 | 0.15 |  | -0.007 | -0.007 |  | Green |
|  | M | 2012 | H117732 | 0.37 | 0.28 |  | -0.037 | -0.028 |  | Blue |
|  | M | 2012 | H44866 | 0.66 | 0.53 |  | -0.024 | -0.031 |  | Red |
|  | F | 2013 | H117935 | 0.24 | 0.40 |  | -0.022 | -0.026 |  | Yellow |
|  | F | 2013 | H121128 | 0.20 | - |  | -0.038 | - |  | Purple |
|  | F | 2013 | H121752 | 0.13 | 0.13 |  | -0.006 | -0.009 |  | Orange |

Data on NDVI were obtained from U.S Geological Survey Famine Early Warning System network (<http://earlywarning.usgs.gov/fews/index.php>) and we used the median NDVI values over the months October to February as a proxy of general conditions and the difference between median NDVI values in October and February (ΔNDVI= NDVI_Feb_ - NDVI_Oct_) as an index of their variation.
